# Growth hormone as concomitant treatment in severe fibromyalgia associated with low IGF-1 serum levels. A pilot study

**DOI:** 10.1186/1471-2474-8-119

**Published:** 2007-11-30

**Authors:** Guillem Cuatrecasas, Cristina Riudavets, Maria Antònia Güell, Albert Nadal

**Affiliations:** 1 Servicio de Endocrinología y Nutrición, Centro Médico Teknon, Vilana 12, E-08022 Barcelona, Spain; 2 Psicología Clínica, Centro Médico Teknon, Vilana 12, E-08022 Barcelona, Spain; 3 Servicio de Reumatología, Centro Médico Teknon, Vilana 12, E-08022 Barcelona, Spain

## Abstract

**Background:**

There is evidence of functional growth hormone (GH) deficiency, expressed by means of low insulin-like growth factor 1 (IGF-1) serum levels, in a subset of fibromyalgia patients. The efficacy of GH versus placebo has been previously suggested in this population. We investigated the efficacy and safety of low dose GH as an adjunct to standard therapy in the treatment of severe, prolonged and well-treated fibromyalgia patients with low IGF-1 levels.

**Methods:**

Twenty-four patients were enrolled in a randomized, open-label, best available care-controlled study. Patients were randomly assigned to receive either 0.0125 mg/kg/d of GH subcutaneously (titrated depending on IGF-1) added to standard therapy or standard therapy alone during one year. The number of tender points, the Fibromyalgia Impact Questionnaire (FIQ) and the EuroQol 5D (EQ-5D), including a Quality of Life visual analogic scale (EQ-VAS) were assessed at different time-points.

**Results:**

At the end of the study, the GH group showed a 60% reduction in the mean number of tender points (pairs) compared to the control group (p < 0.05; 3.25 ± 0.8 *vs*. 8.25 ± 0.9). Similar improvements were observed in FIQ score (p < 0.05) and EQ-VAS scale (p < 0.001). There was a prompt response to GH administration, with most patients showing improvement within the first months in most of the outcomes. The concomitant administration of GH and standard therapy was well tolerated, and no patients discontinued the study due to adverse events.

**Conclusion:**

The present findings indicate the advantage of adding a daily GH dose to the standard therapy in a subset of severe fibromyalgia patients with low IGF-1 serum levels.

**Trial Registration:**

NCT00497562 (ClinicalTrials.gov).

## Background

Fibromyalgia is a common syndrome of non-articular origin, characterized basically by fatigue and widespread musculoskeletal pain, along with tiredness and sleep disturbances. Pain in at least 11 of 18 trigger point sites on digital palpation is the only condition required to fulfil the American College of Rheumatology (ACR) criteria for fibromyalgia [1]. It is thought that fibromyalgia affects between 2.4–3.4% of the adult population, mainly women [2,3].

The cause of fibromyalgia is not known. However, the identification of this syndrome as a single clinical entity has improved the knowledge of its pathophysiology. There is evidence that the syndrome is influenced by psychiatric conditions such as anxiety or depression [4]. Additionally, reduced levels of biogenic amines, increased concentrations of excitatory neurotransmitters (both in the central nervous system nuclea and the medullary dorsal horn) [5], and alterations of the autonomic nervous system and hypothalamic-pituitary-adrenal (HPA) axis have also been described [6].

There is some evidence of functional GH deficiency, expressed as low insulin-like growth factor 1 (IGF-1) serum levels, in a subset of fibromyalgia patients [7]. There is a marked decrease in spontaneous integrated GH secretion [8]. Normal pituitary responsiveness to exogenously administered growth hormone releasing hormone (GHRH) is counteracted by hypersomatostatinemia, as evidenced by the pyridostigmine test [9]. Futhermore, serum GH together with normal/low IGF-1 levels have been found elevated fibromyalgia syndrome, suggesting a GH resistance, and mediating a pro-inflammatory state responsible in part for the pain suffered by these patients [10].

Current consensus based on clinical trials establishes that the best available fibromyalgia treatment should include drugs, rehabilitation and psychological support. Therapies with proven efficacy in fibromyalgia includes tricyclic antidepressants, selective serotonin reuptake inhibitors (SSRI), and opioid analgesics [11]. Regarding fibromyalgia patients with low IGF-1 levels, GH administration demonstrated efficacy and a good tolerability profile in a placebo-controlled study [12]. Although other studies allowed the presence of other medications, no clinical trials have been conducted with GH as an adjuvant agent added to homogeneous active treatment in the management of severe and prolonged fibromyalgia with low IGF-1 levels so far.

## Methods

### Patients and study design

Women greater than 18 years old with severe fibromyalgia and abnormally low IGF-1 levels included in an exercise rehabilitation and psychological program and stable under standard intensive treatment for at least 6 months participated in this prospective, randomized, open-label, best available treatment-controlled clinical trial. All patients fulfilled the 1990 ACR diagnostic criteria [1] and had an IGF-1 level <250 ng/mL (or 1 standard deviation bellow the mean value corresponding to age and gender according to laboratory reference values). Other inclusion criteria were duration of fibromyalgia for 1 year or greater, pain in at least 16 (8 bilateral) of the 18 trigger points and a score in the Fibromyalgia Impact Questionnaire (FIQ) ≥ 75.

Exclusion criteria were as follows: Disabling physical or mental status; previous or current malignancies, either active or inactive; intracranial space occupying lesion; any relevant endocrine disorder including diabetes mellitus; history of another pituitary disorder; previous treatment with growth hormone; other systemic or joint inflammatory rheumatic conditions; and known to be hypersensitive to somatropin or any of the excipients. Pregnant women, nursing mothers, or women with childbearing potential not using adequate contraceptive methods were also excluded.

This study was conducted in the Centro Médico Teknon between May 2004 and November 2005. The study was conducted in accordance with the Declaration of Helsinki and received the local institutional review board and Spanish Drug Agency (n°C03-0456) approvals. It has been registered (NCT00497562) at ClinicalTrials.gov. All patients gave written informed consent prior to their inclusion in the study.

### Treatment administration

The study medication is a human growth hormone produced by recombinant DNA technology in a mammalian cell line (Saizen^® ^8 mg click easy, solution for injection). Patients were randomly assigned, according to a computer-generated randomisation table, to receive either 0.0125 mg/kg/d of r-hGH subcutaneously added to standard intensive therapy or standard intensive therapy alone during one year. Standard intensive therapy (administered under stable conditions for at least 6 months before inclusion) consisted of three oral administered drugs: amitriptyline 10–50 mg/d, fluoxetine (or other SSRI 10–40 mg/d) and tramadol 100–400 mg/d. These doses were individually adjusted according to the associated pathology in each case. Doses of r-hGH were adjusted after the first month according to IGF-1 plasma levels and/or the appearance of adverse events possibly related to GH.

### Study Procedures

Prior to starting treatment administration, medical history, physical examination, selection criteria and informed consent were recorded. Additionally, an insulin tolerance test (ITT) and a cranial magnetic resonance imaging (MRI) were performed at baseline visit at investigator's discretion when IGF-1 levels were < 150 ng/mL (or 2 standard deviations below the mean value corresponding to age and gender according to laboratory reference values).

Follow-up visits were scheduled at 1, 3, 6 and 12 months. At baseline and follow-up visits, investigators determined the number of tender points according to the ACR criteria for fibromyalgia and patients self-completed the FIQ, the EQ-5D, and a health-related Quality of Life Visual Analogic Scale (VAS). A blood sample was drawn at baseline and at follow-up visits to measure blood cell count, biochemical profile and other laboratory assays including GH, IGF-1, thyroid-stimulating hormone (TSH), thyroxine (free T4), triiodothyronine (free T3), Estradiol (E2), progesterone (P4), urinary free cortisol, insulinemia, apolipoprotein B, C-reactive protein, erythrocyte sedimentation rate, rheumatoid factor, aldolase and creatinkinase. Adverse events, concomitant medication and treatment compliance were recorded at each follow-up visit. A flow chart of the study procedures is shown in Table 1.

**Table 1:** Study flow-chart

	Visit 1 baseline	Visit 2 1 month	Visit 3 3 months	Visit 4 6 months	Visit 5 12 months
**Data**					
Medical history	X				
Informed consent	X				
Demographic data	X				
Inclusion criteria	X				
Adverse events		X	X	X	X
Concomitant medication		X	X	X	X
Treatment compliance		X	X	X	X
Physical examination	X			X	X
MRI (1)	X				
Laboratory assays	X	X	X	X	X
IGF-1 levels (2)	X	X	X	X	X
Insulin tolerance secretion test (3)	X				
Tender points evaluation	X	X	X	X	X
Fibromyalgia Impact questionnaire	X	X	X	X	X
EuroQol EQ-5D + VAS	X	X	X	X	X
Rehabilitation	X	X	X	X	X
Psychological assistance	X	X	X	X	X

(1) When baseline IGF-1 < 150 ng/ml to exclude pituitary lesion.

(2) For patient inclusion and r-hGH dose adjustment.

(3) When IGF-1 < 150 ng/ml.

### Efficacy Assessments

The primary efficacy endpoint was the reduction in the number of tender points at 12 months with respect to baseline. Fibromyalgia trigger points were assessed by an observer blind to the treatment group using the protocol described by Wolfe et al. employing 18 (9 bilateral) standardized sites and according to ACR criteria [1]. Secondary efficacy endpoints included the number of patients who discontinued the study due to lack of efficacy, and the completion of spanish validated versions of FIQ [13] and the EQ-5D Quality of Life questionnaire [14] at baseline and follow-up visits. The FIQ is a 10-item instrument with good reliability and validity, that measures physical impairment, wellbeing, missed work, pain, fatigue, rest, stiffness, anxiety and depression [15]. The EQ-5D consists of two parts; the Self-Questionnaire, which captures respondent's descriptions of health problems on a 5-dimensional classification of mobility, self-care, usual activities, pain/discomfort and anxiety/depression, and the EQ-VAS, which is a 20-centimeter visual analogue scale where the respondent rates her health state today between 0 (worst imaginable) and 100 (best imaginable). The EQ-5D has been shown to be both reliable and valid in adult patients with GH deficiency but also in multiple musculoskeletal diseases [16].

### Laboratory assays

Serum GH, IGF-1, TSH, free T4, free T3, E2, P4 and insuline levels were determined by automated chemiluminescent immunoassay system using a commercially available kit (IMMULITE^® ^2000, DPC, Los Angeles, CA). The interassay coefficient of variation of GH and IGF-1 assays was 4.52% and 3.04%, respectively. The analytical sensitivity of GH and IGF-1 assays was 0.05 ng/mL and <25 ng/mL, respectively. Serum cortisol levels were also determined by chemiluminiscence using the Liason Analizer (Sorin Diagnostics S.pA, Italy).

### Safety

Safety was assessed by the investigator through physical examinations, including vital signs, haematological and biochemical laboratory tests, reporting of adverse events, and local tolerability. In order to minimize the occurrence of adverse events, r-hGH doses were individually adjusted during the study according to age-adjusted IGF-1 serum levels (+ 2 standard deviation corresponding to 450 ug/dL) and the investigator discretion.

### Statistics

This was a pilot study with an exploratory goal. Thus, a sample size was estimated to obtain preliminary results that would allow evaluation for further possible investigations. Quantitative endpoints are presented as mean and standard deviation (SD) or median and range. The 95% confidence interval was used to indicate the precision of an estimate. The homogeneity of variances was analysed by a Levene's test and the within-group comparisons employed t test and the non-parametric Mann-Whitney Wilcoxon test when necessary. Categorical data are presented as absolute numbers and percentages. A Chi-Square analysis or Fisher exact-test were used to compare these variables when applicable.

Regarding the main variable, the differences between groups were analyzed by analysis of covariance (ANCOVA), with basal number of tender points as a covariate. The time course within-group comparisons were analysed by repeated measurements analysis of variance (ANOVA). All p values were based on a two-tailed distribution, and 5% level of significance was considered. The statistical analysis was based on Intention to Treat. The SPSS statistical software package (version 12.0) was used for statistical analysis.

## Results

The study population included 24 women with a mean age of 48.5 years, randomly assigned to r-hGH (n = 12) or control (n = 12) groups. All patients fulfilled the ACR criteria for fibromyalgia, the cut-off levels for severity (16/18 positive trigger points and FIQ>75) and duration of symptoms (>1 year), IGF-1 levels>250 ug/dL (three patients in the control group had IGF-1<150 ug/dL and undergone an ITT with a peak GH response above 5 ug/dL and had normal MNR) and completed the study. GH levels were normal and comparable in both groups. Baseline data are shown in Table 2.

**Table 2:** Baseline characteristics of 24 patients included in the study.

Data, mean (SD)	r-hGH (n = 12)	Control (n = 12)	p value t student test
Demographics			
Age, years	47,4 (7,6)	49,6 (9,4)	0.555
Weight, kg	72,9 (12,3)	66,0 (9,4)	0.153
Waist, cm	89.2 (10.3)	76.6 (3.2)	0.01
BMI, Kg/m^2^	25,1 (5,3)	162,2 (5,9)	0.334
Fibromyalgia symptoms duration (prior study), months	21 (6)	23 (7)	0,204
Vital signs			
Systolic blood pressure, mm Hg	124,8 (15,4)	117,5 (10,3)	0.222
Diastolic blood pressure, mm Hg	80,8 (6,6)	74,2 (7,9)	0.052
Heart rate, beats/min	75,8 (6,4)	77,9 (11,8)	0.721
Laboratory data			
GH, ng/mL	0,4 (0,3)	0,9 (0,8)	0.134
IGF-1 g/L	173.3 (49.4)	98.6 (36.0)	<0.001
TSH, μU/mL	1.3 (0.9)	1.3 (0.9)	0.998
free T4, ng/mL	3.3 (4.9)	5.5 (6.9)	0.433
free T3, pg/mL	3.7 (0.7)	2.9 (0.5)	0.041
Estradiol E2, pg/mL	117.8 (17.7)	98.4 (81.7)	0.708
P4, ng/mL	0.4 (0.3)	0.7 (0.3)	0.287
Urinary free cortisol, μg/24 h	98.2 (66.1)	44.5 (11.9)	0.030
Insulinemia U/dL	11.9 (14.7)	6.0 (2.7)	0.306
Apolipoprotein B, mg/L	102.8 (26.7)	98.7 (66.1)	0.744
C-Reactive protein, mg/L	3.2 (0.4)	3.5 (0.0)	0.417
Erythrocyte sedimentation rate, mm	12.8 (5.8)	11.0 (4.6)	0.482

**Figure 1:**
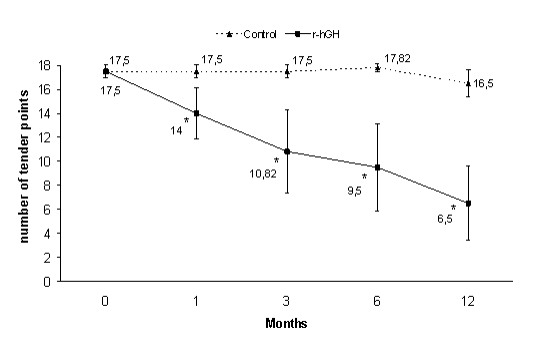
**Mean (CI) number of tender points**. *p < 0.05 vs control group (ANCOVA); p = 0.001 time course within-group comparisons (repeated measures ANOVA) (statistical analysis performed with values expressed as pairs of tender points).

**Figure 2:**
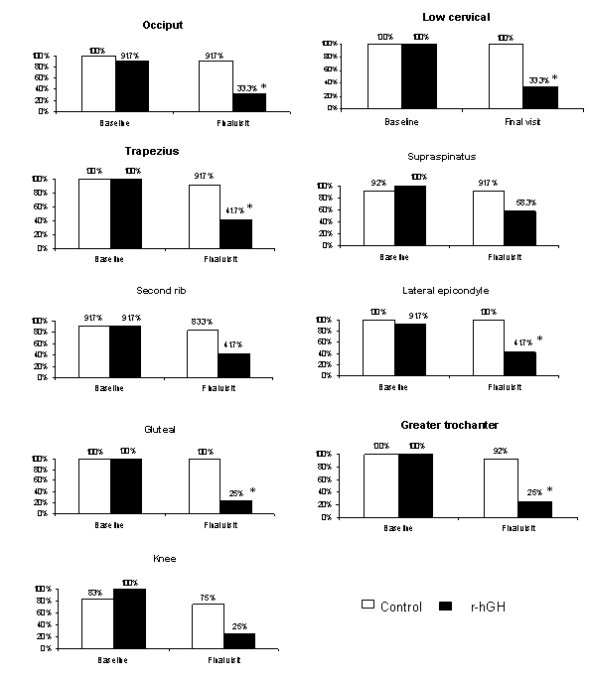
**Percentage of patients with pain in each of the 9 bilateral tender point sites**. *p < 0.05 vs control group (chi-square test).

With regard to the primary efficacy outcome, the study showed a reduction in the number of tender points (pairs) in the r-hGH group compared to the control group, at 12-month evaluation (p = 0.0001) (Figure 1). The mean (SD) number of baseline tender points (pairs) was 8.75 (0.4) in both treatment groups, and 3.25 (2.7) and 8.25 (0.9) in r-hGH and control groups at 12 months, respectively. Statistically significant between-group differences were also observed at 1, 3 and 6-month evaluations (p < 0.05) (Figure 1). Patients receiving r-hGH also had a within-group improvement in the number of tender points (pairs), achieving statistical significance at all the follow-up visits compared to baseline (p = 0.001). The control group failed to achieve a significant within-group improvement between baseline and 12-month evaluation. Figure 2 shows improvement in pain in each of the 9 bilateral trigger point sites after 12 months for each treatment group.

**Figure 3:**
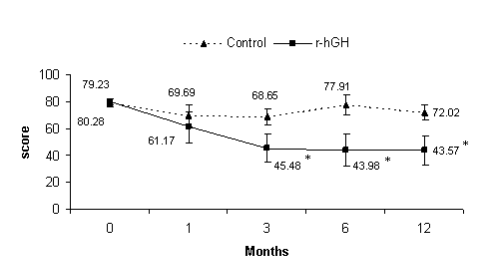
**Mean (CI) total score of Fibromyalgia Impact Questionnaire**. *p < 0.05 vs control group (ANCOVA); p = 0.001 time course within-group comparisons (repeated measures ANOVA).

Regarding the secondary endpoints, there were significant differences between treatments favouring the r-hGH group for the total FIQ score at 3, 6 and 12 months (p < 0.05) (Figure 3). The mean (SD) total FIQ score was 80.28 (4.0) and 79.23 (5.1) at baseline, and 43.57 (19.0) and 72.02 (9.0) at 12-month evaluation in the r-hGH and control groups, respectively. Statistically significant improvements compared to baseline appeared from month 3 until the end of the study in the r-hGH group (p = 0.001).

A statistical significant improvement was also observed in r-hGH group (p < 0.05) in comparison to control group in both pain and fatigue FIQ subscales. Thus, for the FIQ-pain subscale, the mean (SD) was 8.67 (0.98) and 8.5 (1.09) at baseline, and 5.17 (2.44) and 7.75 (1.36) at 12-month evaluation in the r-hGH and control groups, respectively. A similar pattern of results was observed for the FIQ-fatigue subscale, with a mean (SD) of 8.75 (1.6) in both groups at baseline, and 4.92 (1.8) and 7.75 (1.2) at 12-month evaluation in r-hGH and control groups, respectively.

**Figure 4:**
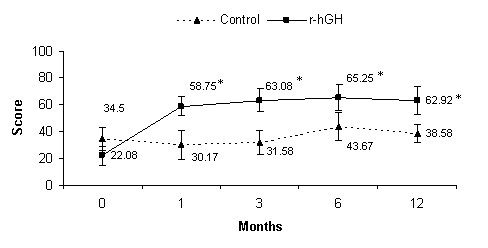
**Mean (CI) score of EQ-VAS**. *p < 0.05 vs control group (ANCOVA); p = 0.001 time course within-group comparisons (repeated measures ANOVA).

There were also significant differences between treatment groups in terms of Quality of Life (EQ-5D). Thus, there was a significant improvement in the mean VAS score for the r-hGH group compared to control group at 1, 3, 6 and 12 months (p < 0.05), with a within-group significant improvement from month 1 until the end of the study compared to baseline (p = 0.001) (Figure 4). With regard to the EQ-5D questionnaire, a similar trend was observed in the mean weighted score with significant differences between treatment groups at 1 and 3 months (p < 0.05) and within-group significant improvement in the r-hGH group from month 1 to the end of follow-up compared to baseline (p = 0.017). The mean (SD) weighted score was -0.04 (0.3) and 0.13 (0.2) at baseline, 0.79 (0.1) and 0.19 (0.4) at month 1, 0.74 (0.2) and 0.34 (0.4) at month 3, and 0.62 (0.3) and 0.47 (0.3) at 12 month evaluation in the r-hGH and control groups, respectively. It is worth mentioning that there were differences between groups at baseline in both EQ-VAS and EQ Self-Questionnaire, with worse self-reported quality of life scores in the r-hGH group compared to control group.

No withdrawals due to lack of efficacy were reported in the study.

A statistically significant correlation between the increase of IGF-1 serum levels and clinical improvement was observed at the 1^st ^month (p = 0.028). No further correlation was observed with IGF-1 or any other biochemical marker.

In terms of tolerability, no important changes in any of the clinical or laboratory parameters were observed in any of the treatment groups. No withdrawals due to adverse events were reported in the study in any treatment group. Despite the high r-hGH dosage (mean dose 0,123 mg/Kg/d; range 0,8 mg- 1,4 mg), only 10 adverse events were reported in 4 patients, all of them in the r-hGH group (two cases each of joint pain, oedema and anemia and 1 case each of carpal tunnel syndrome, cholelithiasis, hypertriglyceridemia and hyperglycemia). No patient discontinued the study due to adverse events. All reported adverse events were considered to be possibly related to the study treatment. All events were of mild intensity and no serious adverse events were reported during the study.

## Discussion

Fibromyalgia is a common syndrome of widespread musculoskeletal pain. Although its etiology is not known, there is evidence that fibromyalgia is influenced by several factors, including alterations of autonomic nervous system activity and HPA axis [6]. Growth Hormone secreted by the pituitary acts on the liver and other tissues to stimulate the production of IGF-1 [17]. Despite IGF-1 levels are influenced by age, gender, nutrition, body mass index, chronic stress and concomitant psychiatric medication, and a low sensibility stays (between 37% and 48%, when standardized by age and gender for a controlled population) [18], a subset of fibromyalgia patients with low IGF-1 serum levels (cut-off 150 ug/dL for -2SD, as in our study) but with normal insulin tolerance test has been identified [7]. We hypothesize that this could be caused by a low integrated GH secretion [8] together with a hypersomatostatinergic tone [9].

The standard pharmacological therapy for this syndrome includes the combination of tricyclic antidepressants, SSRI and opioid analgesics [11]. Currently in fibromyalgia patients with low IGF-1 serum levels, the administration of r-hGH demonstrated efficacy as compared with placebo in a randomized, double-blind study [12]. However, although other studies permitted the use of other medications, no homogeneous active-controlled GH add-on studies have been performed to date in this population. Taking into account this evidence, it seemed interesting to conduct a study that evaluates the efficacy and safety of low r-hGH doses added to standard therapy in the treatment of severe (FIQ>75 and 16/18 positive trigger points) and prolonged (>1 year) fibromyalgia patients with low IGF-1 serum levels.

The present study is a prospective, randomized, open-label, best available treatment-controlled clinical trial. Even though no placebo group was included, the different overall response observed between treatment groups, as well as the intra-group differences in the r-hGH group as compared with baseline, supports the internal validity of this assay. The endpoints used in this study are well-validated outcome measures in the evaluation of fibromyalgia. The manual trigger point examination (in this case by an observer blinded to the treatment) has been historically considered a key feature in the definition of fibromyalgia 6. Likewise, the FIQ and EQ-5D have been widely used in the clinical evaluation of this syndrome and GH deficiency, respectivelly [3,16]. Other associated symptoms such as fatigue were evaluated with individual FIQ subscales. The one-year duration of this study is longer than the previous GH controlled trial (9 months) 12, thus avoiding the seasonal variability of fibromyalgia symptoms. At the end of the study (12 months evaluation), the mean number of tender points in the r-hGH group showed a reduction of 60% compared to the control group and 63% compared to baseline (p < 0.05 in both comparisons). Similar results were observed at the same time-point assessment in the FIQ, with a mean score reduction of 39.5% compared to control group and 45.7% compared to baseline (p < 0.05 for both comparisons). This global FIQ score improvement in the GH group is the result of a similar improvement in all the specific subscales (fatigue, pain, professional activity, tenderness, anxiety, depression, morning weakness, global well-being, laboral absenteeism, and physical dysfunction).

Regarding the quality of life self-assessment, the VAS evaluation showed a similar trend, with a 38.7% mean score improvement at 12 months in the r-hGH group versus control group and 64.9% versus baseline (p = 0.001 in both comparisons). The weighted score assessment of the EQ-5D questionnaire at the end of the study compared to baseline showed a mean change of 0.59 and 0.33 in r-hGH and control groups, respectively. This change from baseline achieved statistical significance in the r-hGH group (p = 0.017).

It is important to mention the prompt response in the r-hGH group, evidenced and maintained since the first month assessment. In our study all 12 patients in the r-hGH group experienced global improvement and 1 patient even scored 100 in the VAS scale at the end of the study. This contrasts favourably with Bennett's previous study [12] where only 15 out of 22 patients in the GH treatment group experienced improvement. The difference in success rates could possibly be explained by the greater basal homogeneity in the present study regarding duration and severity (FIQ ≥ 75; >16/18 positive trigger points) of disease, active and concomitant pharmacological treatment, together with active rehabilitation and psychological assistance. The lack of correlation between IGF-1 and markers of clinical improvement may be due in part to the step-down r-hGH dose adjustments performed in order to avoid adverse events, and in part due to the unexpected statistical difference seen in IGF-1 levels. These differences in a small number group are due to 3 patients in the control group with values lower than 150 ug/dL (who had an ITT and MNR for this reason). IGF-1 was considered as a dyctomic variable at inclusion, not as a continuous one, therefore we cannot expect it to be a predictor marker of response.

The concomitant administration of r-hGH and standard therapy was well tolerated. No important changes in any of the clinical or laboratory parameters were observed in the two groups. A total of 10 adverse events were reported in 4 patients, all of them in the r-hGH group. However, only one (8.3%) carpal tunnel syndrome was described in comparison with the 7 cases (31.8%) reported in the GH treatment group in Bennett's previous study [12] despite the fact that both administered 0.0125 mg/kg/d of r-hGH. There was 1 case of hyperglycemia at the 1^st ^month follow-up vist, which was normalized at the 3^rd ^month evaluation after reducing the dose by 0.02 mg/d. No patients discontinued the study due to r-hGH related adverse events. No control group dropouts were seen, possibly due to the intense standard treatment received (pharmacological, psychological and rehabilitation).

## Conclusion

The present study suggests the advantage of adding a 0.0125 mg/Kg r-hGH daily dose to the standard therapy versus the standard therapy alone in a subset of severe fibromyalgia patients with low IGF-1 serum levels. Obviously, the results of this study should be viewed in light of the methodological limitations, such as the open assignment to the study drug, the lack of a placebo group and the small sample size. However, the consistency and magnitude of these results, obtained in this well-characterized sample and with long-term treatment period, added to the similar trend observed in the previous placebo-controlled study, are encouraging and warrant further validation in a multicenter, double-blind confirmatory study which is currently ongoing.

## Competing interests

The authors have received external funding from Serono-Iberia (now Merck España S.L) for the preparation of study data for submission. The authors declare that they have no other competing interest.

## Authors' contributions

GC and AN designed the study, made the overall coordination and prepared the data for presentation. Both GC and CR visited the patients in the endocrinological view. CR passed the EQ-5D questionnaire and trained the patients to the use of GH. GC made the blind randomization, up or down-titrate the GH dose according to IGF-1 or adverse effects (clinical or laboratory). MAG performed the psychological assistance required in trial protocol in both groups. AN visited the patients in the reumatological view, assessed the tender points and administered the VAS and FIQ scale and subscales (blinded to the treatment groups) and also evaluated the fibromyalgia standard therapy dosage. All authors have read and approved the final manuscript.

## Pre-publication history

The pre-publication history for this paper can be accessed here:


